# PREX1 improves homeostatic proliferation to maintain a naive CD4^+^ T cell compartment in older age

**DOI:** 10.1172/jci.insight.172848

**Published:** 2024-02-08

**Authors:** Huimin Zhang, Hirohisa Okuyama, Abhinav Jain, Rohit R. Jadhav, Bowen Wu, Ines Sturmlechner, Jose Morales, Shozo Ohtsuki, Cornelia M. Weyand, Jӧrg J. Goronzy

**Affiliations:** 1 Department of Immunology,; 2 Department of Medicine, Division of Rheumatology, and; 3 Robert and Arlene Kogod Center on Aging, Mayo Clinic, Rochester, Minnesota, USA.

**Keywords:** Aging, Immunology, Adaptive immunity, T cells

## Abstract

The human adult immune system maintains normal T cell counts and compensates for T cell loss throughout life, mainly through peripheral homeostatic proliferation after the ability of the thymus to generate new T cells has rapidly declined at adolescence. This process is mainly driven by STAT5-activating cytokines, most importantly IL-7, and is very effective in maintaining a large naive CD4^+^ T cell compartment into older age. Here, we describe that naive CD4^+^ T cells undergo adaptations to optimize IL-7 responses by upregulating the guanine-nucleotide exchange factor PREX1 in older age. PREX1 promotes nuclear translocation of phosphorylated STAT5, thereby supporting homeostatic proliferation in response to IL-7. Through the same mechanism, increased expression of PREX1 also biases naive cells to differentiate into effector T cells. These findings are consistent with the concept that primarily beneficial adaptations during aging, i.e., improved homeostasis, account for unfavorable functions of the aged immune system, in this case biased differentiation.

## Abstract 2

In the aged immune system, naive CD4^+^ T cells are selected for increased expression of PREX1 that endows them with enhanced IL7 responsiveness.

## Graphical abstract

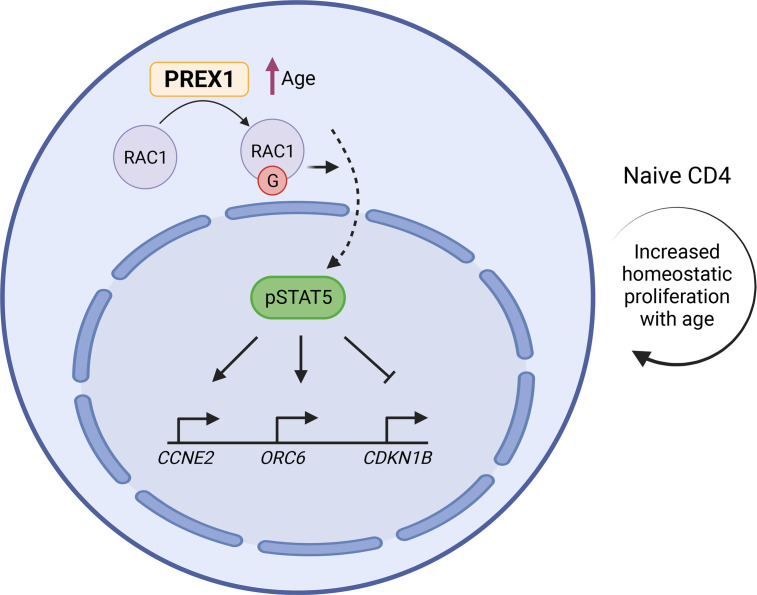

## Introduction

Loss of regenerative capacity is one universal hallmark of aging (1). Originally described for hematopoietic stem cells that lose self-renewal potential as they encounter DNA damage or enter differentiation pathways, it is pertinent for many organ systems, including T cells (2, 3). Novel T cells expressing unique T cell receptors (TCRs) are exclusively generated in the thymus; however, thymic output already declines in late childhood and particularly during puberty; the bulk of T cell generation during the adult lifetime derives from division of existing peripheral T cells. It has been estimated that less than 20% of T cell generation already in young adults is of thymic origin, which further declines to 1% or less in older adults (4, 5). Remarkably, mechanisms maintaining a naive CD4^+^ T cell compartment are very effective in contrast with naive CD8^+^ T cells (6).

Replenishing T cell loss by homeostatic proliferation has the advantage of not having to rely on a complex thymic selection machinery that may be prone to error with increasing age; instead, it can build on an existing, highly selected, and diverse repertoire of T cells generated during early life. However, it also brings about several challenges. Because no T cells with new TCR rearrangements are inserted into the peripheral T cell compartment, cumulative loss of entire T cell progenies sharing a TCR sequence could lead to a contraction in TCR repertoire diversity. Computational simulation of T cell homeostasis has shown that massive contraction indeed can occur, however, only in the setting of major peripheral selection forces (7). In healthy older adults, we observed a contraction by a factor of 2 to 5, which, given the immense diversity of the human TCR repertoire, is unlikely of functional importance (8, 9). In longitudinal studies, the greatest age-related TCR repertoire attrition was observed in naive CD8^+^ T cells, while gain in clonal expansions was typical for memory CD8^+^ T cells, suggesting that regulation of homeostatic proliferation is subset specific and age-related changes are more profound for CD8^+^ than CD4^+^ T cells (6, 10, 11).

Like stem cells, naive T cells are kept in a quiescent state, with low metabolic and low proliferative activity to preserve longevity. Homeostatic proliferation requires quiescence exit and cell activation, which needs to be controlled to prevent DNA damage as well as the initiation of differentiation programs (12). Indeed, age-associated changes in the chromatin structure mimic the epigenetic signature of T cell differentiation, with increased accessibility to bZIP family transcription factors (TFs) such as BATF and AP1 (13). Again, changes are more prominent in naive CD8^+^ than CD4^+^ T cells (14, 15). In contrast, clear evidence of DNA damage responses or cellular senescence in the transcriptome or epigenome of aged naive T cells is lacking (14, 16). In summary, virtual differentiation through homeostatic proliferation appears to be the major driver of the aging signature in human T cells.

Maintenance of the naive T cell pool is dependent on constant stimulation by extrinsic factors that include recognition of self-peptide–MHC complexes through the TCR and stimulation by growth- and survival-promoting cytokines (17). IL-7 produced in the stromal cell network in secondary lymphoid organs has a central role in homeostatic proliferation by activating the JAK/STAT5 and PI3K/AKT pathways. To survive and expand, naive T cells compete for resources and space in tissues, which eventually should lead to a fitness selection of T cells in response to a changing environment during aging. Indeed, studies have shown a strong skewing in the murine CD8^+^ T cell pool of unimmunized mice due to peripheral selection (18). Moreover, newly produced T cells derived from thymic reconstitution have difficulty in being seeded in the periphery in older mice (19, 20). Such a fitness selection might change the composition of naive T cells with age and be particularly evident in single-cell studies.

Here, we performed single-cell multiomic sequencing and found an age-associated epigenomic signature suggestive of quiescence exit. Differential analysis of single-cell RNA-seq (scRNA-seq) and assay for transposase-accessible chromatin with sequencing (ATAC-seq) data of naive CD4^+^ T cells from young and older adults identified *PREX1* as one top age-associated gene score. PREX1 protein expression was tripled in naive CD4^+^ T cells from older adults. We further showed that PREX1 induced cell cycle gene programs through facilitating nuclear localization of phosphorylated STAT5 (p-STAT5). Correspondingly, PREX1 increased homeostatic proliferation of naive CD4^+^ T cells and promoted effector T cell differentiation after T cell activation. Taken together, our results show that increased expression of PREX1 in older adults is a consequence of fitness selection that is important to maintain naive T cell compartment sizes in humans.

## Results

### Age-associated gene signatures of quiescence exit in naive CD4^+^ T cells.

**Figure 1:**
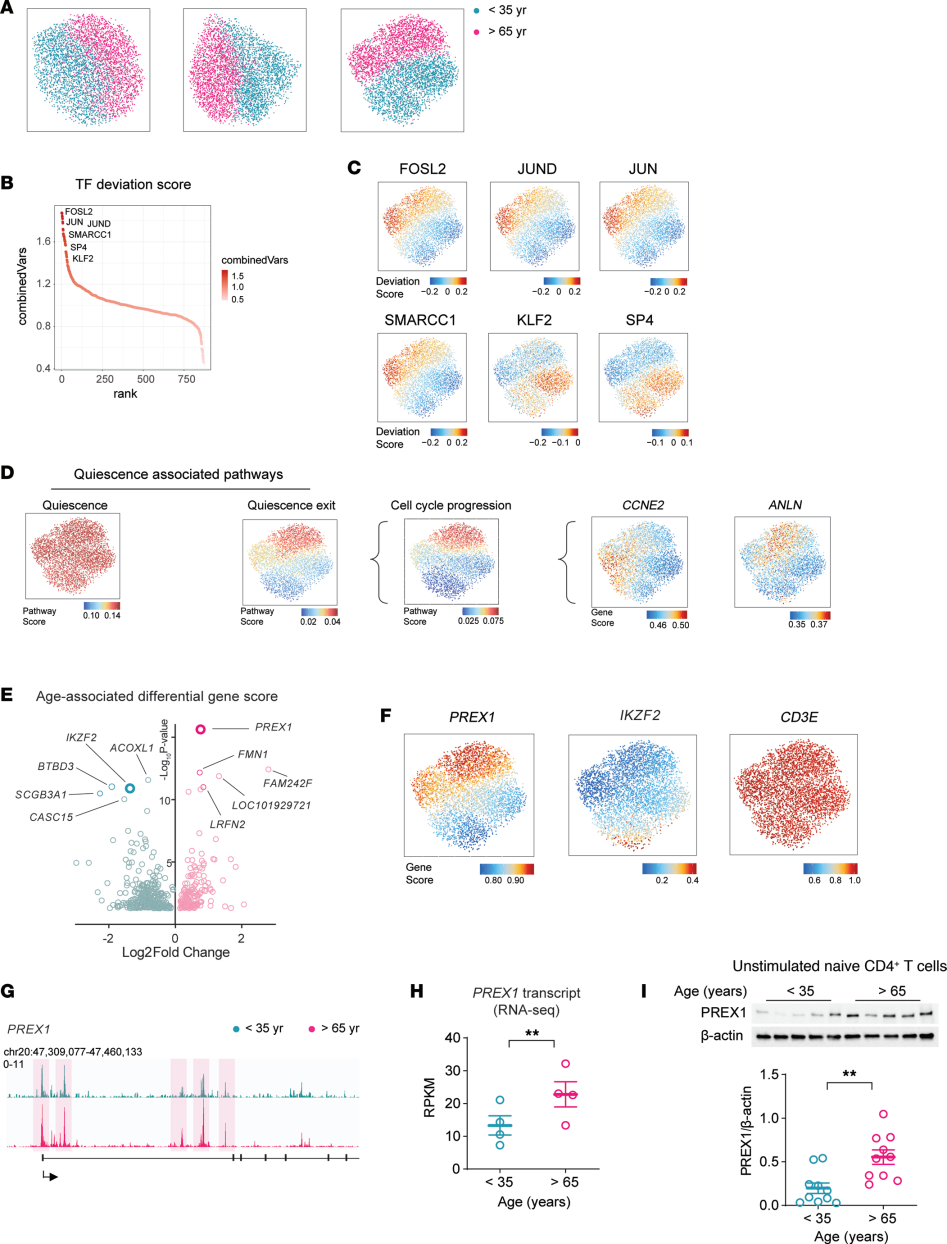
Naive CD4^+^ T cells from older adults have a quiescence exit gene signature. (**A**) UMAPs generated from scATAC-seq data, scRNA-seq data, and combined data sets of peripheral naive CD4^+^ T cells from 2 young individuals (cyan) and 2 older individuals (magenta). (**B**) Ranking of ChromVar transcription factor (TF) deviation scores from scATAC-seq data. TFs ranking at the top are indicated. (**C**) TF deviation scores projected on UMAP space of integrated scRNA-seq/scATAC-seq data. (**D**) Quiescence-associated pathways or gene scores were projected onto integrated UMAPs. Pathway definition followed the gene grouping criteria described in Cheung and Rando (25). (**E**) Volcano plot of gene scores from scATAC-seq data comparing cells from young and older adults. Cyan color indicates genes with more accessible regulatory sites in cells from young adults and magenta in those from older adults. Bolded circles highlight some of the top differential genes. (**F**) Gene score of indicated genes projected on the integrated UMAP space. (**G**) Genome tracks of the *PREX1* promoter region. Magenta shades highlight those peaks more open in older adults. (**H**) *PREX1* transcript levels from bulk RNA-seq. RPKM, reads per kilobase per million mapped reads. Data presented as mean ± SEM. Significance was assessed by 2-tailed, paired Student’s *t* test. (**I**) PREX1 immunoblot of naive CD4^+^ T cells from 5 young and 5 older adults (upper panel). Summary of PREX1 protein expression in naive CD4^+^ T cells from 10 young and 10 older adults (bottom panel). Data presented as mean ± SEM. Significance was assessed by 2-tailed, unpaired Student’s *t* test. **P < 0.01.

To identify age-associated gene signatures in human naive CD4^+^ T cells, we performed combined single-nuclei ATAC-seq and RNA-seq on unstimulated naive CD4^+^ T cells from young and older adults, isolated by negative selection as described in the Methods. Projections on the uniform manifold approximation and projection (UMAP) space are shown for individual as well as the combined RNA- and ATAC-seq data sets (Figure 1A). Cells of older adults were clearly shifted from those of the young, most obviously seen for the integrated UMAP (Figure 1A), suggesting a global age-associated signature, i.e., the entire naive CD4^+^ T cell compartment undergoes reorientation with age. To characterize the spectrum of the epigenetic organization in an unbiased manner, we employed chromVAR to infer the TFs whose binding motifs are enriched at accessible chromatin regions (Figure 1B). Among the TFs with highest binding motif enrichment were the AP-1 and KLF/SP superfamilies. AP-1 members, including FOS, FOSL2, JUN, and JUND, are related to T cell activation and exit of quiescence, raising the possibility that the signature is caused by a contamination with non-naive T cells that increases with age. Reacquisition of a naive phenotype by memory cells is more frequently observed for CD8^+^ than CD4^+^ T cells (21–23), and such cells accumulate with age. Purity of the isolated naive CD4^+^ T cell population was generally greater than 95%, as determined by staining for CD3, CD4, CD45RA, and CCR7 (Supplemental Figure 1A; supplemental material available online with this article; https://doi.org/10.1172/jci.insight.172848DS1). A more detailed cytometric analysis showed a small population of CD95^+^CD4^+^ stem-like memory T cells in older adults, but no other phenotypic markers of cell activation or differentiation (Supplemental Figure 1B). In contrast with this low frequency, deviation scores of the AP-1 family when projected on the integrated UMAP showed universally higher binding-motif enrichments in naive CD4^+^ T cells from older adults (Figure 1C). Conversely, KLF2, required for restraining naive T cell activation (24), showed binding-motif enrichment in naive CD4^+^ T cells from young adults (Figure 1C). SP4, another member of the KLF/SP superfamily, had a similar enrichment pattern to that of KLF2. The TF motif enrichment distribution suggested that the age-associated epigenomic changes largely involve regulatory programs of T cell activation and cellular quiescence. Cheung and Rando provided a comprehensive summary of the stem cell quiescence-regulatory networks by extracting the common quiescent stem cell signature from 3 types of stem cells, including hematopoietic stem cells (25). We projected the enrichment scores for their pathways of downregulated genes (quiescence exit) and upregulated genes (quiescence) on the integrated UMAPs (Figure 1D). Scores for the quiescence pathway were universally high irrespective of age, consistent with the naive state of CD4^+^ T cells. In contrast, the quiescence exit pathway showed a preferential enrichment in the naive CD4^+^ T cells from older adults (Figure 1D). Subcategorization of the quiescence exit pathway revealed that the genes functioning in cell cycle progression mostly accounted for the upregulation in cells from older adults (Figure 1D). Particularly, *CCNE2* (coding for cyclin E2 and required for G_1_/S phase transition) and *ANLN* (coding for anilin and required for mitosis) are more open at older age. Interestingly, *CCNE2* and *ANLA* are distributed in largely different populations of cells, suggesting that the naive CD4^+^ T cells in older individuals captured in this single snapshot are undergoing different stages of proliferation. Thus, the TF binding motif analysis and the pathway enrichment analysis collectively indicate that naive CD4^+^ T cells from older adults have undergone epigenomic changes that favor cell cycle progression.

### Age-associated upregulation of PREX1 in naive CD4^+^ T cells.

We performed differential analysis of gene scores (computed accessibility within the extended gene body and weighted distal regulatory regions from scATAC-seq) and transcripts (from scRNA-seq) comparing naive CD4^+^ T cells from young and older adults. Transcript analysis identified 126 genes that were differentially expressed with age. Comparison of gene scores from single-cell multiomic data sets identified 504 genes and was therefore more sensitive, possibly due to including differentially poised genes in a resting cell population (Figure 1E). *PREX1* ranked among the most significantly more accessible genes in older adults, coding for the guanine-nucleotide exchange factor (GEF) for RAC1, which is known to function in cancer cell proliferation and metastasis (26). Other more accessible genes were *LRFN2* and *SNED1*, both containing a fibronectin type III domain related to cell adhesion. The more open genes in young adults included *IKZF2*, *BTBD3*, and *SCGB3A1*. We have recently described that lower expression of *IKZF2* in naive CD4^+^ T cells from older adults caused accelerated T cell differentiation after activation by amplifying STAT5 signaling (27). UMAP projection of *IKZF2* and *PREX1* gene scores showed that the abundance of each gene was down- or upregulated across all cells from older adults, whereas the constitutively expressed gene *CD3E* was uniformly distributed irrespective of age (Figure 1F), suggesting that clustering was not biased by the total transcript input of each cell. To confirm that the increased gene score for *PREX1* is not due to contaminating CD95^+^ T cells, we examined the single-cell data for the coexpression of *PREX1* and *TNFRS6* (encoding CD95). No correlation was observed (Supplemental Figure 1C). Moreover, histograms showed that the expression of *PREX1* followed a unimodal distribution.

To verify differential accessibility with age, we analyzed bulk ATAC-seq data of unstimulated naive CD3^+^CD4^+^CD45RA^+^CCR7^+^ T cells from another 4 young and 4 older individuals (27). Tracks shown in Figure 1G show areas of differential *PREX1* accessibility. Bulk RNA-seq data confirmed the higher abundance of *PREX1* transcripts in older adults (Figure 1H). We examined PREX1 protein expression in an additional 10 young and 10 older adults and found PREX1 protein levels to be tripled in naive CD4^+^ T cells from older adults (Figure 1I).

In our previous studies, we found that age-associated changes in chromatin accessibility of naive and central memory T cells reflect those seen with differentiation, presumably due to the inability to maintain quiescence with homeostatic proliferation. Of note, this subtle differentiation is population-wide and not limited to a small subset of cells (15, 16). To determine whether PREX1 expression is also driven by differentiation despite the negative results with stem-like memory cells, we reanalyzed a large single-cell transcriptomic data set of peripheral CD4^+^ T cells (28). As shown in Supplemental Figure 2A, transcription of *PREX1* is clearly differentiation dependent, with the lowest expression in naive and the highest in CD4^+^ effector memory T cells re-expressing CD45RA (Temra cells). However, short-term culture with IL-7 was not sufficient to induce PREX1 expression (Supplemental Figure 2B). Moreover, after TCR stimulation, *PREX1* transcripts declined until 6 hours and did not recover by 48 hours (Supplemental Figure 2C). We conclude that neither IL-7 nor TCR acutely upregulates PREX1. However, based on the higher expression in memory cells, increased expression of PREX1 with age in naive cells may be reflective of partial differentiation, as occurs over time in vivo.

### PREX1 upregulates cell cycle gene programs through facilitating STAT5 nuclear translocation.

**Figure 2:**
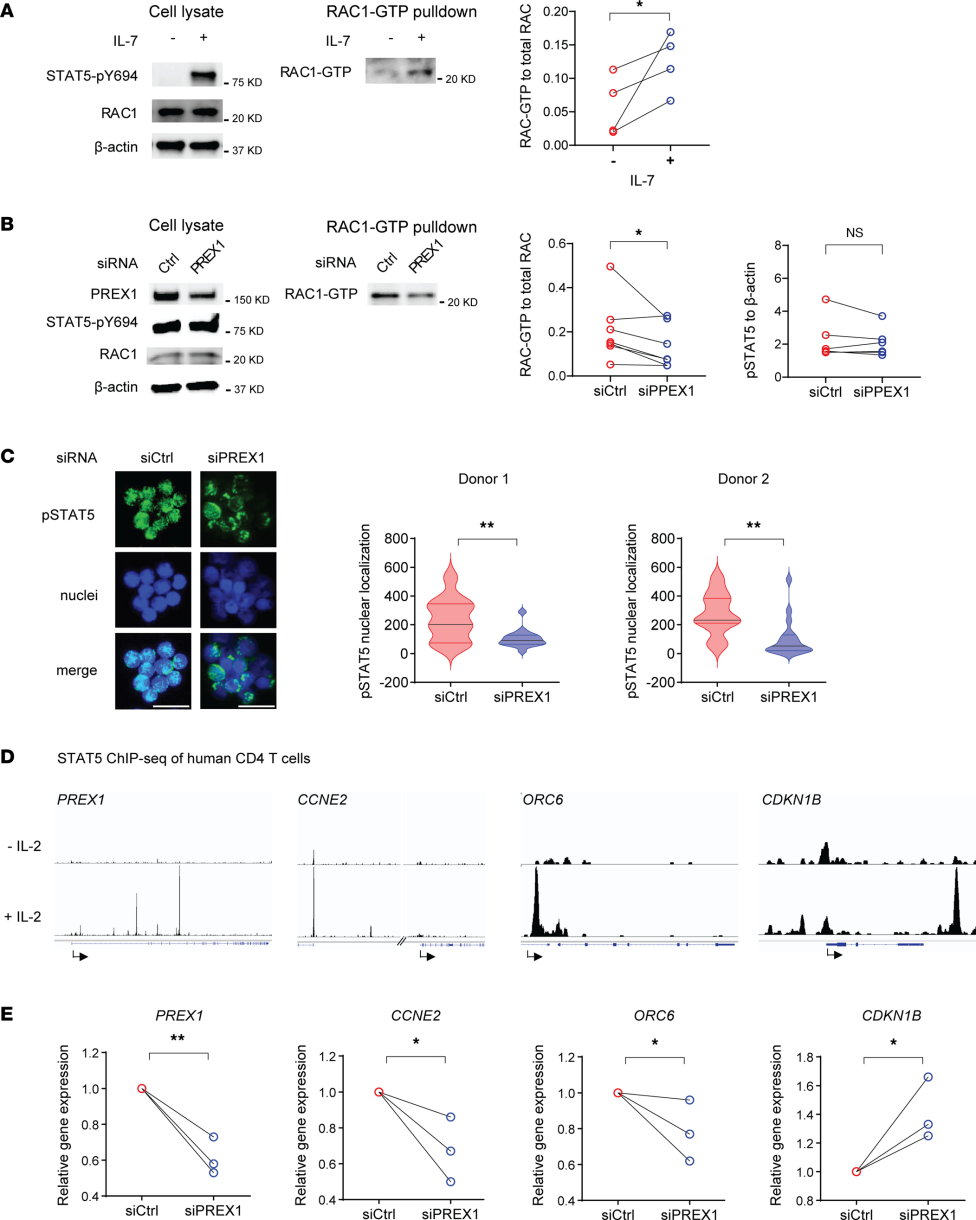
PREX1 upregulates cell cycle gene programs through facilitating STAT5 nuclear translocation. (**A**) RAC1-GTP and STAT5 phosphorylation of naive CD4^+^ T cells from 4 older adults cultured in the presence or absence of IL-7 (5 ng/mL) for 15 minutes. Representative of 2 experiments. Statistical comparison was done by 1-tailed, paired Student’s *t* test. *P < 0.05. (**B**) Naive CD4^+^ T cells from 7 older adults were transfected with either control or *PREX1* siRNA and assayed for STAT5 phosphorylation and RAC1-GTP. Statistical comparison was done by 1-tailed, paired Student’s *t* test. *P < 0.05. (**C**) Immunofluorescence microscopy images of p-STAT5 (green), nuclei (blue), and merged staining (left panel). Scale bars: 20 μm. Cells from 2 donors were assayed. Data depicted as violin plots showing median and quartiles. Statistical comparison was done for each donor by 2-tailed, unpaired Student’s *t* test. **P < 0.01. (**D**) STAT5 ChIP-seq (32) genome tracks of *PREX1* and indicated cell cycle genes after IL-2 stimulation. (**E**) Relative gene expression of *PREX1* and cell cycle genes after *PREX1* silencing of naive CD4^+^ T cells from 3 older adults and after 12 days of incubation with IL-7 (5 ng/mL). Statistical comparison was done by 1-tailed, unpaired Student’s *t* test. *P < 0.05. **P < 0.01.

*PREX1* encodes the GEF for RAC1, which is well established for its function in cell cycle progression (29). Thus, we speculated that PREX1 plays a critical role in promoting cell cycle regulation in naive CD4^+^ T cells from older adults. We first examined RAC1 activity in unstimulated, negatively selected naive CD4^+^ T cells and found minimal RAC1-GTP in the resting state (Figure 2A). Naive T cell homeostasis requires periodic exposure to the cytokine IL-7 (30). We therefore tested RAC1 activity of naive CD3^+^CD4^+^CD45RA^+^CCR7^+^ T cells after IL-7 exposure and found that IL-7 induced loading of RAC1 with GTP (Figure 2A). To determine whether this IL-7–induced RAC1 activity was mediated by PREX1, we silenced *PREX1* gene expression and found a corresponding downregulation of RAC1-GTP (Figure 2B). RAC1 GTPase activity is required for the nuclear translocation of p-STAT5 (31). Total p-STAT5 was not influenced by PREX1 (Figure 2B), consistent with our finding that PREX1 did not influence the expression of the IL-7 receptor (IL-7RA) (Supplemental Figure 2D). To determine whether nuclear p-STAT5 is regulated by PREX1 in T cells, we examined the cellular distribution and found reduced nuclear localization of p-STAT5 after *PREX1* silencing (Figure 2C). p-STAT5 is known to regulate T cell proliferation and we found STAT5 binding of a number of cell cycle genes in published ChIP-seq data (32), including *CCNE2*, the gene more accessible in naive CD4^+^ T cells from older adults (Figure 2D). Partial *PREX1* silencing downregulated the genes promoting cell cycle progression, *CCNE2* and *ORC6*, in IL-7–driven cultures, while upregulating the inhibitor of cell cycle progression, *CDKN1B* (Figure 2E). We concluded that PREX1 upregulates the cell cycle gene network through facilitating nuclear translocation of active STAT5. Interestingly, we also found STAT5 binding to the *PREX1* promoter (Figure 2D), suggesting that STAT5 is involved in the transcriptional regulation of *PREX1*.

### PREX1 accelerates homeostatic proliferation of naive CD4^+^ T cells.

**Figure 3:**
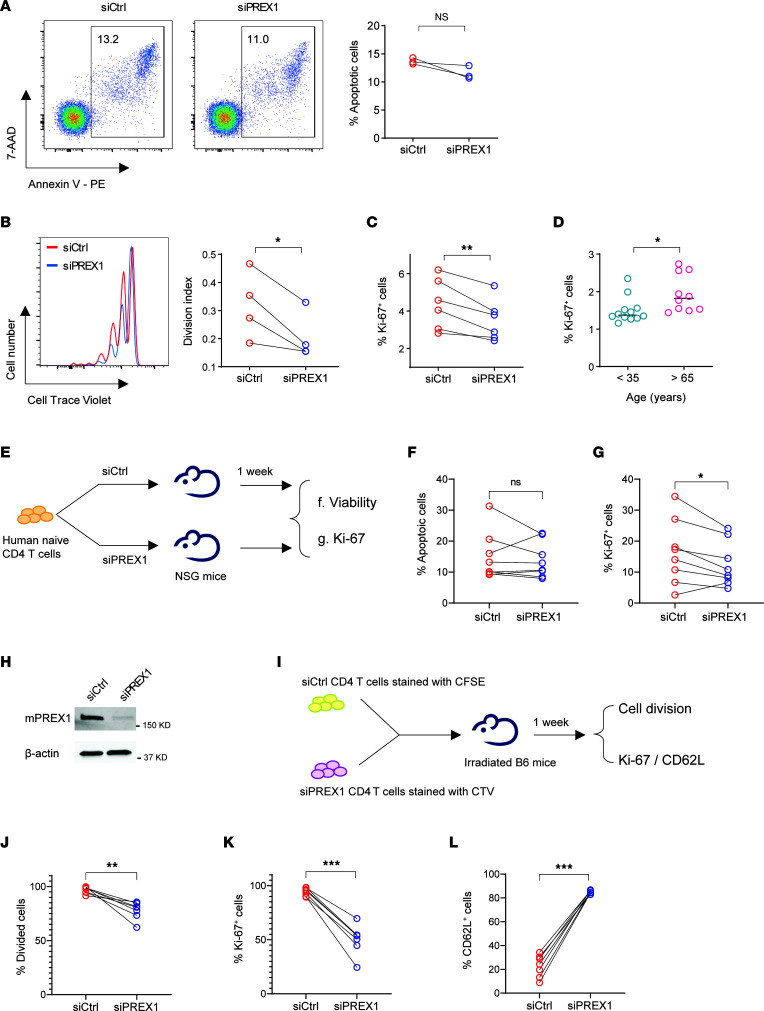
PREX1 accelerates homeostatic proliferation of naive CD4^+^ T cells. Naive CD4^+^ T cells from older adults were transfected with control or *PREX1* siRNA, incubated with IL-7 (5 ng/mL) for 14–20 days, and assayed for (**A**) apoptosis (*n* = 3), (**B**) CellTrace Violet (CTV) dilution (*n* = 4), and (**C**) Ki-67 expression (*n* = 6). Statistical analysis was done by 2-tailed, paired Student’s *t* test. *P < 0.05, **P < 0.01. (**D**) Ki-67 expression in naive CD4^+^ T cells directly ex vivo from 12 young and 10 older adults. Statistical analysis was done by 2-tailed, unpaired Student’s *t* test. *P < 0.05. (**E**) Schematics of in vivo studies. Human naive CD4^+^ T cells from 8 older adults, transfected with siCtrl or siPREX1, were injected into NSG mice. One week after injection, spleens were harvested. (**F** and **G**) Frequencies of (**F**) apoptotic (*n* = 8) and (**G**) Ki-67–expressing (*n* = 8) human CD4^+^ T cells were determined. Statistical analysis was done by 2-tailed, paired Student’s *t* test. *P < 0.05. (**H**) Mouse CD4^+^ T cells were transfected with either control or mouse *Prex1* siRNA and assayed for PREX1 expression. (**I**) Schematics of in vivo studies. CD45.1^+^CD4^+^ T cells transfected with siCtrl or siPREX1 were stained with CFSE or CTV and injected into irradiated B6 CD45.2 mice. One week after injection, spleens were harvested. (**J**–**L**) Frequencies of (**J**) divided cells (*n* = 7), (**K**) Ki-67–expressing CD4^+^ T cells (*n* = 7), and (**L**) CD62L-expressing CD4^+^ T cells (*n* = 7). Statistical analysis was done by 2-tailed, paired Student’s *t* test. **P < 0.01, ***P < 0.001.

To examine the effect of PREX1 on naive T cell homeostasis, we silenced *PREX1* gene expression and monitored naive CD4^+^ T cell proliferation and survival in the presence of IL-7. When *PREX1* was partially silenced in naive CD3^+^CD4^+^CD45RA^+^CCR7^+^ T cells from older adults, IL-7–mediated cell survival was not significantly affected (Figure 3A). However, proliferation was significantly reduced as measured by 2 independent approaches: CellTrace Violet (CTV) dilution (Figure 3B) and Ki-67 staining (Figure 3C). Older adults had higher proliferative activity of naive CD4^+^ T cells ex vivo (Figure 3D), consistent with the upregulation of cell cycle gene programs (Figure 1D) that may result from the increased expression of *PREX1*.

To directly determine whether PREX1 exerts an effect on proliferation in vivo, we silenced *PREX1* in human naive CD4^+^ T cells and transferred the cells into immunocompromised NSG mice (Figure 3E). The *PREX1*-silenced naive CD4^+^ T cells showed robust viability (Figure 3F) yet subdued proliferation (Figure 3G), consistent with the in vitro findings. One limitation of this experimental design is that it is across different species. An alternative mouse model to provide further evidence is leukopenia-induced proliferation, in which proliferation is accelerated by self-MHC recognition and IL-7 (33). We transfected CD4^+^ T cells from B6 mice with control or *Prex1* siRNA (Figure 3H), labeled the cells with either CFSE or CTV, and adoptively transferred them into irradiated autologous mice by tail vein injection (Figure 3I). The number of cells having undergone division as determined by dye dilution or Ki-67 expression was significantly reduced with *Prex1* silencing (Figure 3, J and K). Conversely, *Prex1* silencing protected cells from losing CD62L expression, consistent with reduced effector cell differentiation (Figure 3L). Taken together, these results show that PREX1 accelerates homeostatic proliferation, and its increased expression may account for the higher turnover of naive CD4^+^ T cells in older individuals.

### PREX1 supports effector T cell differentiation.

**Figure 4:**
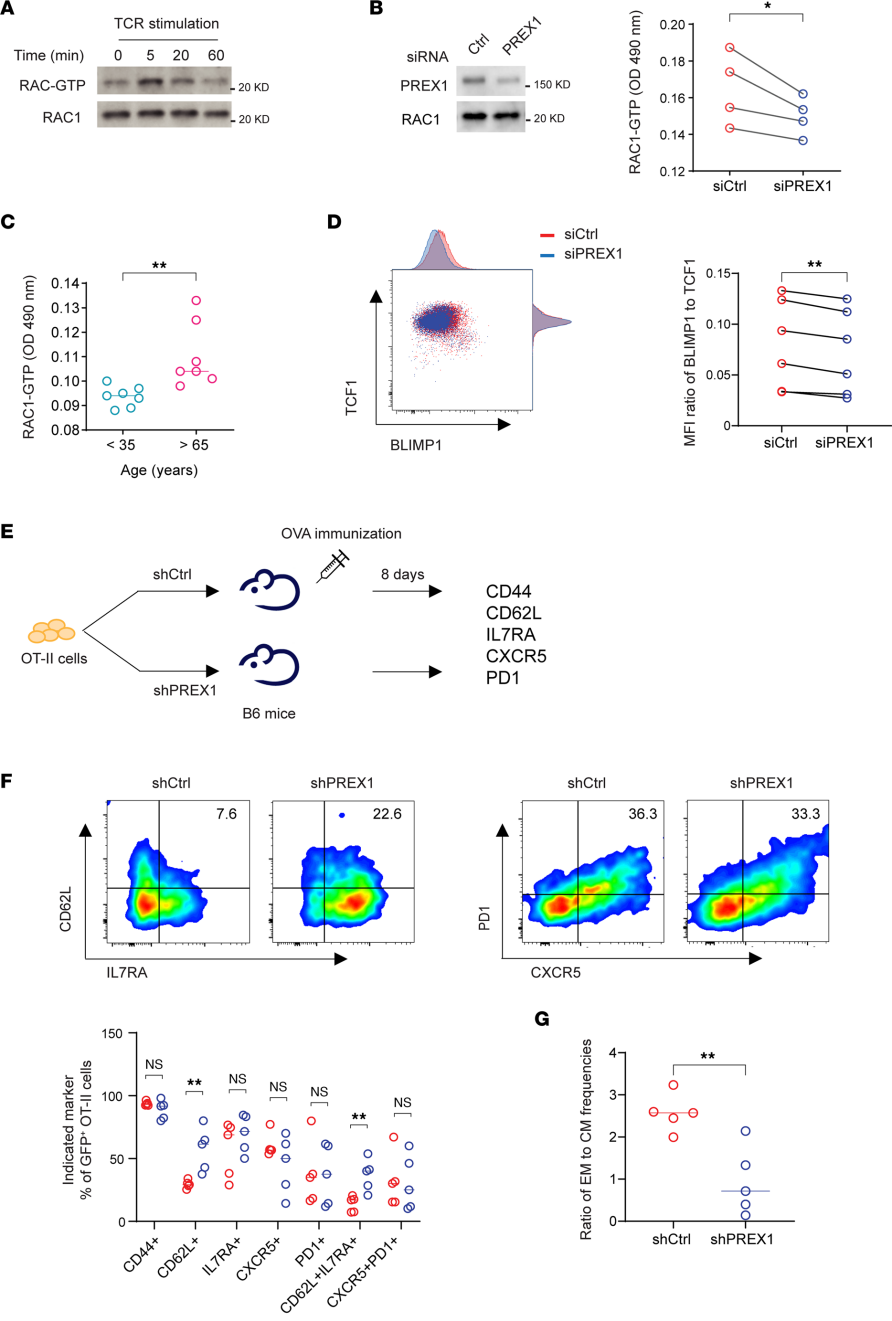
PREX1 facilitates effector T cell differentiation. Naive CD4^+^ T cells were activated with anti-CD3/anti-CD28 antibody–coated polystyrene beads. (**A**) RAC1-GTP was measured at indicated times of activation. (**B**) RAC1-GTP was measured at 5 minutes of activation in control- or *PREX1* siRNA–transfected cells from 4 older adults. Statistical analysis was done by 1-tailed, paired Student’s *t* test. *P < 0.05. (**C**) RAC1-GTP was measured after 5 minutes of activation in naive CD4^+^ T cells from 7 young and 7 older adults. Statistical analysis was done by 2-tailed, unpaired Student’s *t* test. **P < 0.01. (**D**) Naive CD4^+^ T cells from 6 older adults were activated for 5 days after control or *PREX1* silencing. BLIMP1 and TCF1 expression was measured by flow cytometry. Statistical analysis was done by 2-tailed, paired Student’s *t* test. **P < 0.01. (**E**) Schematics of in vivo studies. OT-II naive CD4^+^ T cells transduced with shCtrl or shPREX1 were injected into B6 mice. Eight days after injection, spleens were harvested and CD4^+^ T cells were enriched and stained for indicated markers. (**F**) Frequencies of GFP^+^CD4^+^ T cells transduced with shCtrl (red) or shPREX1 (blue) expressing indicated markers (bottom). Five mice per group. (**G**) Ratio of CD44^+^CD62L^–^ effector memory (EM) to CD44^+^CD62L^+^ central memory (CM) T cells. Five mice per group. Statistical analysis of **F** and **G** was done by 2-tailed, unpaired Student’s *t* test. **P < 0.01.

The finding that PREX1 augmented STAT5 signaling raised the possibility that it has a role in the preferential effector differentiation of naive T cells from older adults after TCR stimulation (27). We first examined RAC1 activity in naive CD4^+^ T cells stimulated with polystyrene beads coated with intermediate amounts of anti-CD3 and anti-CD28 antibodies (27). We observed robust RAC1-GTP generation within 5 minutes of TCR stimulation (Figure 4A). Silencing *PREX1* downregulated TCR-induced RAC1 activity (Figure 4B). Activation-induced RAC1 activity was lower in negatively selected, naive CD4^+^ T cells from younger adults, consistent with their lower expression of PREX1 (Figure 4C). To determine whether PREX1-RAC1 affected CD4^+^ T cell differentiation, we assessed BLIMP1 and TCF1 expression as TFs indicative of effector and memory T cells, respectively. Partial silencing of *PREX1* significantly reduced the induction of BLIMP1 expression after CD4^+^ T cell activation, whereas TCF1 was not affected (Figure 4D). Given the limitation of in vitro systems, we wanted to assess the influence of PREX1 activity on T cell differentiation in vivo. OVA-reactive TCR-transgenic OT-II cells were transduced with shRNA for *Prex1* or control and then adoptively transferred into B6 mice that were subsequently immunized with 4-hydroxy-3-nitrophenylacetyl OVA (NP-OVA) (Figure 4E). *Prex1* silencing shifted the composition of OT-II cells to a central memory phenotype. In particular, the frequency of CD62L^+^IL-7RA^+^ cells was increased. Generation of CXCR5^+^PD-1^+^ cells was not affected (Figure 4, F and G). Taken together, our results show that PREX1 favors effector cell differentiation, and its upregulation with age may contribute to the enhanced effector CD4^+^ T cell differentiation seen in older adults.

## Discussion

Genome-wide RNA-seq and chromatin accessibility studies of purified naive T cells so far have provided insights into how T cells change with age and what drives these changes (34). Most age-associated signatures are reminiscent of cell differentiation, indicating that these changes are part of an adaptation and not a senescence program (14, 16, 35). Whether these signatures are derived from a small subset of T cells has remained unclear. Stem-like memory CD8^+^ T cells frequently contaminate phenotypically defined naive CD8^+^ T cells. In contrast, CD4^+^ T cells, having encountered antigen, maintain their memory phenotype without reverting back to that of naive cells (22). However, we also see an increasing contamination by CD95^+^CD4^+^ T cells in the naive T cell compartment with age (Supplemental Figure 1B). Moreover, Giles et al. identified 14 phenotypic T cell subsets and proposed that age-associated differences arise from shifts in subset distributions (36). Here, we used single-nuclei multiomic sequencing to compare naive CD4^+^ T cells from young and older individuals and to distinguish between global and subset-specific gene signatures in naive T cell aging. Similar to others (36–38), we found that distal accessible chromatin regions from ATAC-seq are superior for distinguishing cell populations than proximal sites or transcriptomic data. For our comparison, we used a gene score, i.e., an accessibility score assigned to each gene based on the entire gene body accessibility, including putative distal regulatory elements while minimizing unrelated regulatory elements. We identified *PREX1* as a top age-associated gene with poised or active gene-regulatory regions. Age-associated increased expression was global and not limited to a subset of more differentiated cells such as T cells expressing CD95. In functional studies, PREX1 supported homeostatic proliferation consistent with the interpretation that increased expression was a consequence of peripheral fitness selection. Specifically, we found that PREX1 promoted cell cycle gene programs through nuclear translocation of p-STAT5 in response to IL-7.

After puberty and progressively so with older age, de novo generation of T cells is markedly reduced, yet a substantial naive CD4^+^ T cell pool is maintained into older age (9). Tsukamoto et al. introduced the concept of peripheral fitness selection as an underlying mechanism of safeguarding a naive T cell compartment. They found that naive CD4^+^ T cells in older mice were more long-lived, in part due to reduced expression of BIM (39). Even in young animals, recent thymic emigrants are not established in the periphery under lympho-replete conditions, suggesting that they are outcompeted by existing T cells, possibly due to increased IL-7–induced BCL2 (40). In our single-nuclei study of human naive CD4^+^ T cells, we did not find a signature for improved survival, but we found one for quiescence exit and cell cycle progression that was due to increased PREX1 expression. This species-specific difference may not be surprising because mice and humans differ in how they maintain the naive T cell compartment. Thymic T cell generation and peripheral T cell survival are more important in the mouse versus homeostatic proliferation in humans (9, 41). Therefore, one would expect fitness selection for human naive T cells to occur at the level of IL-7–induced proliferation. By augmenting p-STAT5 nuclear translocation after IL-7 stimulation, PREX1 promotes cycle gene programs of naive CD4^+^ T cells in older adults. Conversely, the major effect of cytokine-driven proliferation of naive T cells in the mouse is in generating virtual memory cells, mostly described for CD8^+^ T cells (42). Phenotypically, naive CD8^+^ T cells show increased signs of activation of functional and homeostatic programs in older compared with younger animals (43). With increasing age, the majority of murine memory T cells represent such virtual memory cells (44). Virtual memory cells in older mice lose the ability to proliferate in response to TCR signals, but not IL-15, and express transcriptional and phenotypic markers of senescence, rather than exhaustion (43, 45). Since the cytokine IL-15 implicated in addition to IL-7 in virtual memory cell generation also signals through STAT5, it is very possible that the age-associated increase in dysfunctional virtual memory CD8^+^ T cells is regulated by PREX1 activity.

PREX1 was first identified as a GEF for the Rho family GTPase RAC1 in neutrophils (46). It is critical in RAC1-mediated cell motility and growth in breast cancer (47). It is currently unknown what leads to the increased expression of PREX1 in naive T cells with age. PREX1 expression is higher in memory than naive CD4^+^ T cells, suggesting that the increased expression in naive T cells represents subtle differentiation, as seen with many age-associate signatures. ATAC-seq identified an area of increased accessibility in older adults in the gene body of *PREX1* at coordinates chr20:47377143–47377153 (GRCh37, Figure 1G). In ChIP-seq, this region binds p-STAT5 (Figure 2D) (32). However, in in vitro cultures neither IL-2 nor IL-7 was sufficient to induce *PREX1* transcription, suggesting a need for a cooperative activity of several signaling pathways (Supplemental Figure 2). STAT5 signaling is increased in naive CD4^+^ T cells from older adults due to the aberrant expression of CD25 (27). Upregulation of *PREX1* with age might involve an additional positive feedback loop amplifying the IL-7/p-STAT5/PREX1 pathway.

The success in maintaining a stable compartment size through improved homeostatic proliferation ensures the host an adequate number of naive T cells to fight new pathogens. However, selection of T cell clones with increased STAT5 signaling may also have unfavorable consequences. We have shown in in silico simulations that disproportionate clonal selection under homeostatic proliferation can lead to repertoire contraction and even to an abrupt collapse in TCR diversity (7). Indeed, large naive T cell clones and increased inequality of clonal sizes are observed in older individuals (8), possibly leading to biased antiviral or autoimmune responses. Moreover, when undergoing increasing homeostatic proliferation, naive T cells are challenged with maintaining epigenomic stemness and are at risk of entering differentiation and losing plasticity. We have previously shown that naive T cells from older adults preferentially differentiate into effector T cells high in the expression of BLIMP1 rather than memory T cells and that this effect is in part conferred by increased STAT5 activation early during T cell differentiation (27). Here, we found that naive CD4^+^ T cells from older adults produced more RAC1-GTP upon TCR stimulation and that reducing PREX1 expression attenuated BLIMP1 expression. Collectively, we propose that PREX1 expression is a T cell–intrinsic control mechanism of homeostatic proliferation that is associated with fitness selection of adaptive T cell clones. The obvious benefit for the aging host is a more stable naive CD4^+^ compartment, but at the cost of higher sensitivity to IL-2–mediated signals and associated impact on T cell differentiation (48–50).

## Methods

### Sex as biological variable.

Our study examined male and female participants, and similar findings were obtained for both sexes.

### Study design.

Peripheral blood naive CD4^+^ T cells from 8 healthy individuals were examined by bulk ATAC-seq and RNA-seq; 4 healthy individuals participated in the single-cell multiomic sequencing experiment with approval of the Stanford University and Mayo Clinic Institutional Review Boards (27). For experiments not involving genomic sequencing, samples were collected from leukocyte reduction system (LRS) columns of deidentified donors (*n* = 90, age 21 to 35 years or over 65 years) through the Stanford and Mayo Clinic Blood Bank. Mice were used for experimentation at 6–14 weeks of age. Mice of both sexes were used. All mice were housed in the Mayo Clinic Institutional Animal Facility.

### ATAC-seq and RNA-seq.

ATAC-seq and RNA-seq data were acquired and analyzed as previously reported (27). Briefly, 50,000 cells were collected for generating ATAC-seq and 100,000 cells for generating RNA-seq libraries. ATAC-seq reads were processed, filtered, and mapped to the hg19 genome. RNA-seq reads were processed and mapped to genes in the GRCh37 genome. The experiments were performed in 4 batches, each consisting of 1 young and 1 older individual. To eliminate the batch effects, batching was included in the model as a covariate before performing statistical tests. The differential accessibility peaks or transcripts between groups were estimated by fitting contrasts to the model followed by a robust empirical Bayes moderation and by the Benjamini-Hochberg procedure to control for false discovery rate.

### Single-cell multiome.

Single-cell data were from a previously published data set, in which TCR-stimulated and unstimulated naive CD4^+^ T cells were combined and subjected to nuclei isolation (27). A total of 10,000 nuclei from young donors and 10,000 from older donors were used for library generation following the 10× Genomics Chromium Next GEM Single Cell Multiome ATAC and Gene Expression User Guide and sequenced with Illumina NovaSeq 6000. The scRNA-seq and scATAC-seq data were processed with CellRanger ARC and ArchR (version 1.0.1) (51). To exclude ex vivo– and in vitro–activated cells from the data analysis, CD69^–^ cells were selected and subsetted based on iterative latent semantic indexing dimensionality reduction and graph clustering. The gene score was calculated by integrating peaks from the extended gene body, gene boundary, and bidirectional exponential decay from the transcription start site in ArchR. The score is an accessibility score assigned to each gene based on the entire gene body accessibility, including putative distal regulatory elements while minimizing unrelated regulatory elements. The calculated scores were projected on the UMAP, integrating scATAC-seq and scRNA-seq data. TF motif deviation scores were calculated using ChromVar in ArchR, and pathway enrichment scores using the Ucell package (version 1.1.1) in R (52). Significant differences in gene expression or gene scores between cells from old and young donors were determined by Wilcoxon’s test, with a false discovery rate of 0.05 or less.

### Naive CD4^+^ T cell isolation and PREX1 silencing.

Human peripheral blood mononuclear cells (PBMCs) were isolated from LRS columns by gradient centrifugation. Naive CD4^+^ T cells were isolated with an EasySep Human Naive CD4 T Cell Isolation Kit (STEMCELL Technologies, 19555). Naive CD3^+^CD4^+^CD45RA^+^CCR7^+^ cell purity was higher than 95% (Supplemental Figure 1A). Transfections were performed with either ON-TARGETplus siRNA negative control (Horizon, D-001810-10-20) or siRNA targeting *PREX1* (Horizon, L-010063-01-0010) using the Amaxa Nucleofector system and P3 Primary Cell 4D-Nucleofector X Kit (Lonza, V4XP-3024). Two hours after transfection, cells were resuspended in prewarmed RPMI 1640 medium and cultured for 2–3 days prior to downstream experiments.

### Flow cytometry.

The following antibodies were used for phenotypic characterization: anti-CD3 (BD, 564001), anti-CD4 (BioLegend, 344642), anti-CCR7 (BioLegend, 353225), anti-CD45RA (BD, 555488), anti-CD62L (BioLegend, 304806), anti–IL-7RA (BioLegend, 351365), anti-CD25 (BioLegend, 302635), anti-CD95 (BD, 561978), anti-CD28 (BD, 612815), anti-CD122 (BioLegend, 339009), and Fixable Viability Dye eFluor 780 (Thermo Fisher Scientific, 65-0865-18). For IL-7RA expression, cells were stained with anti-CD3, anti-CD4, anti–IL-7RA (BioLegend, 351340). For proliferation measurements, cells were labeled with CTV (Thermo Fisher Scientific, C34557). Apoptosis was measured with a PE Annexin V Apoptosis Detection Kit (BD, 559763). For Ki-67 staining, cells were fixed (BD, 554655), permeabilized (BD, 558050), and stained with anti–Ki-67 antibody (BD, 561283). For TF staining, cells were fixed (Thermo Fisher Scientific, 00-5123-43), permeabilized with permeabilization buffer (Thermo Fisher Scientific, 00-8333-56), and stained with anti-BLIMP1 (R&D Systems, IC36081A) and anti-TCF1 (Cell Signaling Technology, 14456). Data were acquired with a BD LSR Fortessa and processed with FlowJo v10.

### qPCR.

RNA was isolated with an RNeasy Micro Kit (Qiagen, 74004). Reverse transcription was performed with High-Capacity cDNA Reverse Transcription Kit (Thermo Fisher Scientific, 4368814). Quantitative PCR (qPCR) was carried out using PowerUp SYBR Green Master Mix (Thermo Fisher Scientific, A25776) with the following primers. PREX1-F: GGCATTCCTGCATCGCATC, PREX1-R: CGGGTGTAAACAATACTCCAAGG, CCNE2-F: TAGCTGGTCTGGCGAGGTT, CCNE2-R: ACAGGTGGCCAACAATTCCT, ORC6-F: ACAAGGAGACATATCAGAGCTGT, ORC6-R: AGTGGCCTGGATAAGTCAAGAT, CDKN1B-F: AACGTGCGAGTGTCTAACGG, CDKN1B-R: CCCTCTAGGGGTTTGTGATTCT, ACTB-F: GATCATTGCTCCTCCTGAGC, ACTB-R: CGTCATACTCCTGCTTGCTG, IL7R-F: CCCTCGTGGAGGTAAAGTGC, IL7R-R: CCTTCCCGATAGACGACACTC, mPREX1-F: CGTCTGTGCGTACTCAACGAG, mPREX1-R: CCCAAGTTCGTGCTGAGACTG, mACTB-F: GCTGTATTCCCCTCCATCGTG, and mACTB-R: CACGGTTGGCCTTAGGGTTCAG.

### RAC1 activation assay.

Naive CD4^+^ T cells (4 × 10^6^) were mixed with IL-7 (10 ng/mL) in 100 μL RPMI 1640 medium on ice. For TCR stimulation assays, 2 × 10^6^ naive CD4^+^ T cells were mixed with an equal number of anti-CD3/anti-CD28 antibody–conjugated polystyrene beads (27), briefly centrifuged, and kept on ice. After incubation at 37°C for 10 minutes or indicated times, cells were washed once with 1 mL ice-cold PBS and lysed for RAC1-GTP pull down (Cytoskeleton, BK035).

### Immunoblot.

Cell lysates were resolved in 4%–15% precast TGX gels (Bio-Rad, 4561086). Proteins were transferred to nitrocellulose membranes (Bio-Rad, 1704270), blocked with 5% milk in TBST buffer, and incubated with the following primary antibodies: anti–p-STAT5 (Cell Signaling Technology, 9359S), anti-PREX1 (Cell Signaling Technology, 13168S), anti–β-actin (Cell Signaling Technology, 4970S), and anti-RAC1 antibodies within the RAC1-GTP pull-down kit (Cytoskeleton, BK035). After incubation with secondary antibody (Cell Signaling Technology, 7074 or 7076), membranes were developed for chemiluminescent signals with SuperSignal West Femto Maximum Sensitivity Substrate (Thermo Fisher Scientific, 34095) or Pierce ECL Western Blotting Substrate (Thermo Fisher Scientific, 32106).

### In vivo human T cell proliferation.

Control- and *PREX1*-silenced naive CD4^+^ T cells (3 × 10^6^ cells each) were mixed with 11 × 10^6^ autologous PBMCs depleted of CD4^+^ T cells and intraperitoneally injected into NOD.Cg-*Prkdc^scid^* *Il2rg^tm1Wjl^*/SzJ (NSG) mice that were purchased from the Jackson Laboratory (stock 005557). After 6 days, spleens were harvested and splenocytes were isolated. Cells were blocked with both mouse Fc block (BD, 553142) and human Fc block (BioLegend, 422302). Human CD4^+^ T cells were detected with anti–human CD3 (BioLegend, 300406) and anti–human CD4 (BioLegend, 317426) antibodies and further analyzed for apoptosis and Ki-67.

### Leukopenia-induced T cell proliferation.

C57BL/6 (B6) CD45.1 mice (stock 002014) and B6 CD45.2 mice (stock 000664) were purchased from the Jackson Laboratory. Spleens from B6 CD45.1 mice were homogenized and red blood cells were lysed with ACK lysis buffer (Thermo Fisher Scientific, A1049201). CD4^+^ T cells were isolated with a mouse CD4^+^ T Cell Isolation Kit (Miltenyi Biotec, 130-104-454). Transfections were performed with either ON-TARGETplus siRNA negative control (Horizon, D-001810-10-20) or siRNA targeting *Prex1* (Horizon, L-053658-00-0010) using the Amaxa Nucleofector system and P3 Primary Cell 4D-Nucleofector X Kit. Two hours after transfection, cells were resuspended in prewarmed RPMI 1640 medium and cultured for 2 days. Control and *Prex1*-silenced CD4^+^ T cells were labeled with CFSE (Thermo Fisher Scientific, C34554) and CTV, respectively. Control- and *Prex1*-silenced CD4^+^ T cells (3 × 10^6^ cells each) were mixed and intravenously injected into B6 CD45.2 mice. After 7 days, spleens were harvested and splenocytes were isolated. Cells were blocked with mouse Fc block. CD45.1^+^CD4^+^ T cells were detected with anti-CD45.1 (BioLegend, 110714) and anti–mouse CD4 (BioLegend, 100429) antibodies and further analyzed for proliferation using anti–mouse Ki-67 (BioLegend, 652425) and anti–mouse CD62L (BioLegend, 104408) antibodies.

### Plasmid construct and lentivirus production.

The following short hairpin RNA (shRNA) oligonucleotide sequences were obtained from the RNAi Consortium (TRC, Broad Institute): luciferase shRNA (5′-ATGTTTACTACACTCGGATAT-3′) used as negative control and mPREX1 shRNA1 (5′-AAGGTGCAGCAGTACTATA-3′). Sequences were cloned into the pLKO.3G_X7 vector (Addgene, 171213). Lentiviral particles were produced in HEK-293T cells using Lipofectamine 2000 (Invitrogen, 11668) and lentiviral vector and lentiviral packaging plasmid psPAX2 (Addgene, 12259) and pMD2G (Addgene, 12259). After 48 hours, viruses were harvested by filtration of the HEK-293T supernatant through a 0.45-μm syringe filter. The supernatant including virus was freshly used for transduction.

### Antigen-specific T cell responses in vivo.

Naive CD4^+^ T cells from Tg(TcraTcrb)425Cbn/J OT-II mice (Jackson Laboratory, 004194) were isolated with an EasySep Mouse Naive CD4 T Cell Isolation Kit (STEMCELL Technologies, 19555). Cells were activated in plates coated with anti-CD3 and anti-CD28 antibodies and transduced with virus and polybrene (Sigma-Aldrich, TR-1003-G). The next day, 1 × 10^6^ cells were intravenously transferred into B6 mice. On day 1 after transfer, mice were immunized intraperitoneally with 100 μg NP-OVA (Biosearch, N-5051-10) precipitated in 5% Alum (Sigma-Aldrich, 237086). On day 8 after infection, spleens were harvested and splenocytes were analyzed. Cells were blocked with mouse Fc block. GFP-positive CD4^+^ T cells were detected with GFP fluorescence, anti–mouse TCR (BioLegend, 110714), and anti–mouse CD4, and further analyzed for effector memory/central memory differentiation using anti–mouse CD44 (BioLegend, 103041), anti–mouse CD62L (BioLegend, 104418), anti–mouse IL-7RA (BioLegend, 135023), anti–mouse CXCR5 (BioLegend, 145506), and anti–mouse PD-1 (BioLegend, 135231) antibodies.

### Immunofluorescence.

Control- and *PREX1*-silenced naive CD4^+^ T cells (1 × 10^6^ cells each) were resuspended in 100 μL RPMI 1640 medium containing 10 μg/mL IL-7 and placed in a 37°C water batch for 10 minutes. Cells were fixed (BD, 554655), permeabilized with 0.1% Triton X-100 in PBS, and stained with anti–p-STAT5 (Cell Signaling Technology, 9359S) and Alexa Fluor 488 goat anti–rabbit antibodies (Thermo Fisher Scientific, A-11008). Nuclei were stained with DAPI. Images were acquired with a Zeiss LSM 780 confocal microscope and processed with ImageJ (NIH).

### Statistics.

Statistical analysis was performed using Prism (GraphPad). Paired, 1- or 2-tailed Student’s *t* test or unpaired, 2-tailed Student’s *t* test was used for comparing 2 groups. *P* values less than 0.05 were considered statistically significant.

### Study approval.

Human studies were approved by the Stanford University and Mayo Clinic Institutional Review Boards and participants gave written informed consent. Animal protocols were approved by the Mayo Clinic Institutional Animal Care and Use Committee.

### Data availability.

ATAC-seq, RNA-seq, and single-cell multiomic raw data have been deposited in the NCBI Sequence Read Archive (SRA) with BioProject accession no. PRJNA757466.

## Author contributions

HZ, HO, AJ, CMW, and JJG designed research and interpreted data. HZ, HO, BW, IS, JM, and SO performed experimental work. AJ and RRJ analyzed high-throughput data. HZ, HO, AJ, and JJG wrote the manuscript. The order for co–first authorship was assigned according to the contribution towards the study as follows: HZ initiated the study, developed the concept, and performed experiments. HO performed experiments including all revision experiments. AJ performed bioinformatic analyses throughout the study.

## Supplementary Material

Supplemental data

Unedited blot and gel images

Supporting data values

## Version 1 02/08/2024

In-Press Preview

## Version 2 03/08/2024

Electronic publication
