# Supplementary material for: PREX1 improves homeostatic proliferation to maintain a naive CD4+ T cell compartment in older age
Source: JCI Insight. 2024 Feb 8;9(5):e172848. doi: 10.1172/jci.insight.172848 (PMC10972599; doi:10.1172/jci.insight.172848)
Supplement: Supplemental data [file jciinsight-9-172848-s052.pdf]

Supplemental Material

Supplemental Figure 1

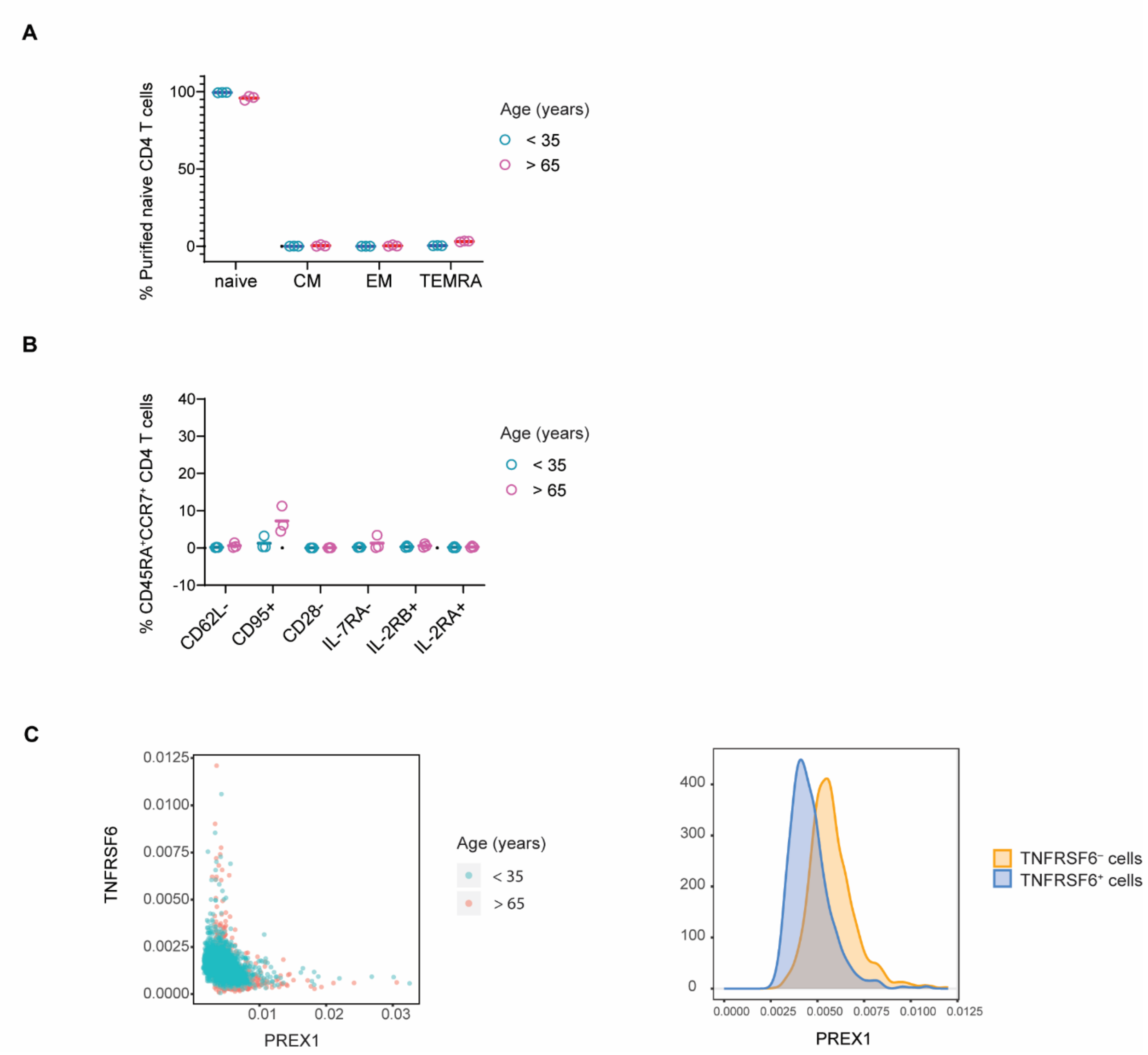

Supplemental Figure 1. Purity of isolated naïve CD4 T cell population. (A) Isolated naïve CD4 T cells from 3 younger and 3 older adults were examined for purity by flow cytometry. (B) Naïve $CD4^+$ $CD45RA^+$ $CCR7^+$ T cells of 3 younger and 3 older adults were compared for the expression of differentiation and activation markers. (C) scRNA transcripts from naïve CD4 T cells of older adults were analyzed for the relationship between *TNFRSF6* (encoding CD95) and *PREX1* transcripts. Data are shown as scatter plots (left) and as histograms of *PREX1* transcripts in single cells expressing or not expressing *TNFRSF6* transcripts (right).

Supplemental Figure 2

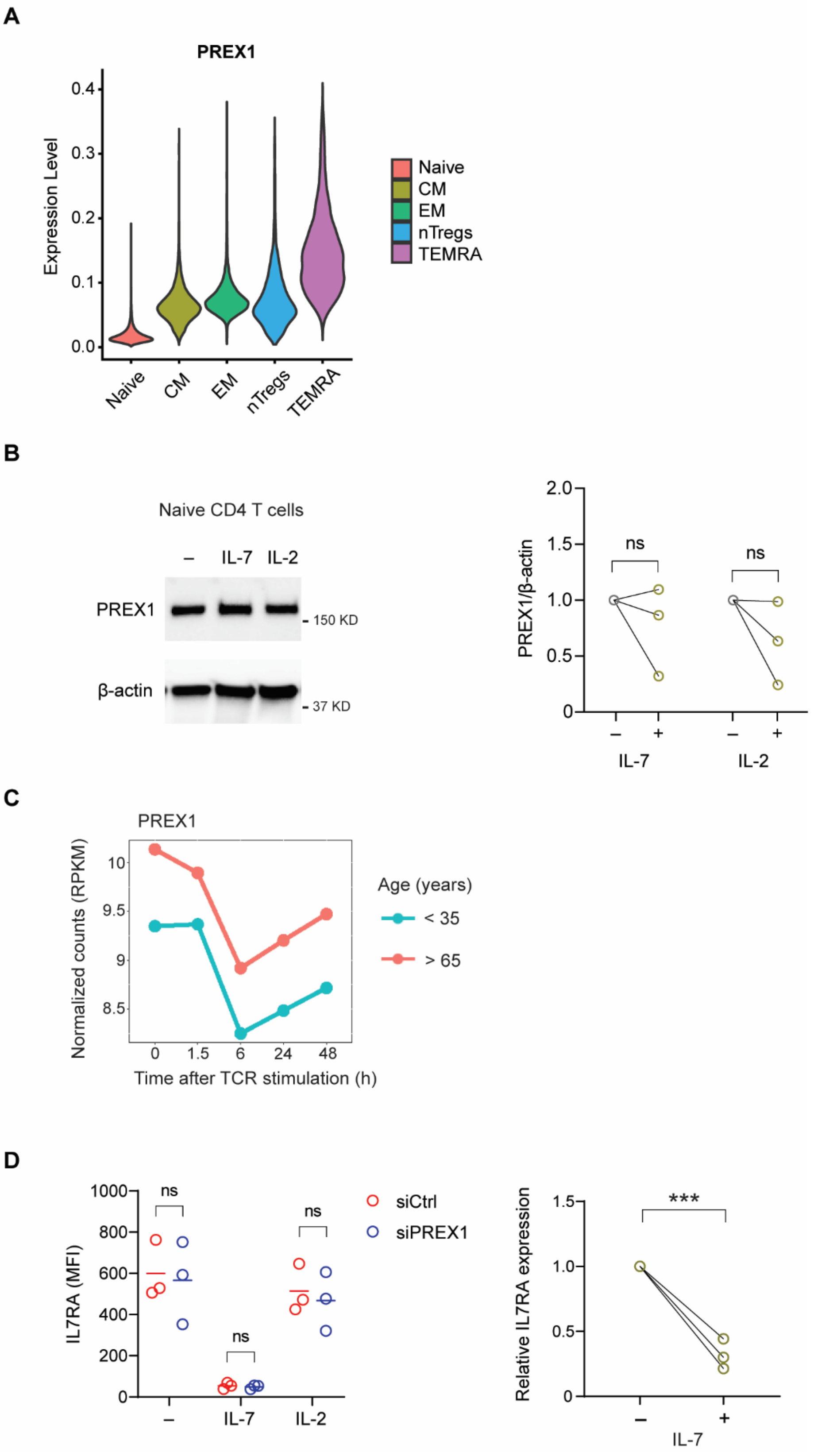

Supplemental Figure 2. CD4 T cell differentiation is associated with increased expression of *PREX1* transcripts. (A) scRNA data downloaded from https://trynkalab.sanger.ac.uk/ were analyzed for the expression of *PREX1* in unstimulated CD4 T cell subsets. Results are shown as violin plots. (B) Naïve CD4 T cells of 3 older adults were cultured with 20 U/mL IL-2 or 10 ng/mL IL-7 for 5 days and then assessed for expression of PREX1. Representative immunoblots (left) and quantitative data from three experiments (right). Statistical analysis was done by two-tailed unpaired t-test. (C) RNA-seq data from BioProject accession no. PRJNA757466 were analyzed for *PREX1* transcripts in naïve CD4 T cells at indicated time points after activation. (D) Naïve CD4 T cells 3 older adults were transfected with siCtrl or siPREX1 and cultured with IL-2 or IL-7. Cells were assessed for the expression of IL7RA by flow cytometry (left). Statistical analysis was done by two-tailed paired t-test. *IL7RA* transcripts were quantified by PCR relative to *ACTB* (right). Statistical analysis was done by two-tailed unpaired t-test.
