# Supplementary material for: PREX1 improves homeostatic proliferation to maintain a naive CD4+ T cell compartment in older age
Source: JCI Insight. 2024 Feb 8;9(5):e172848. doi: 10.1172/jci.insight.172848 (PMC10972599; doi:10.1172/jci.insight.172848)

Full unedited gel for Figure 1I

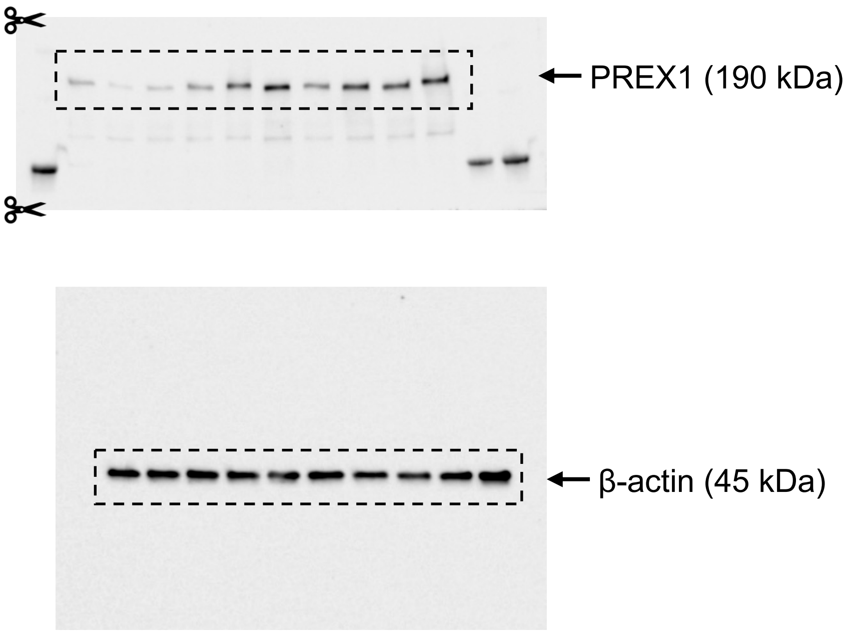

Full unedited gel for Figure 2A

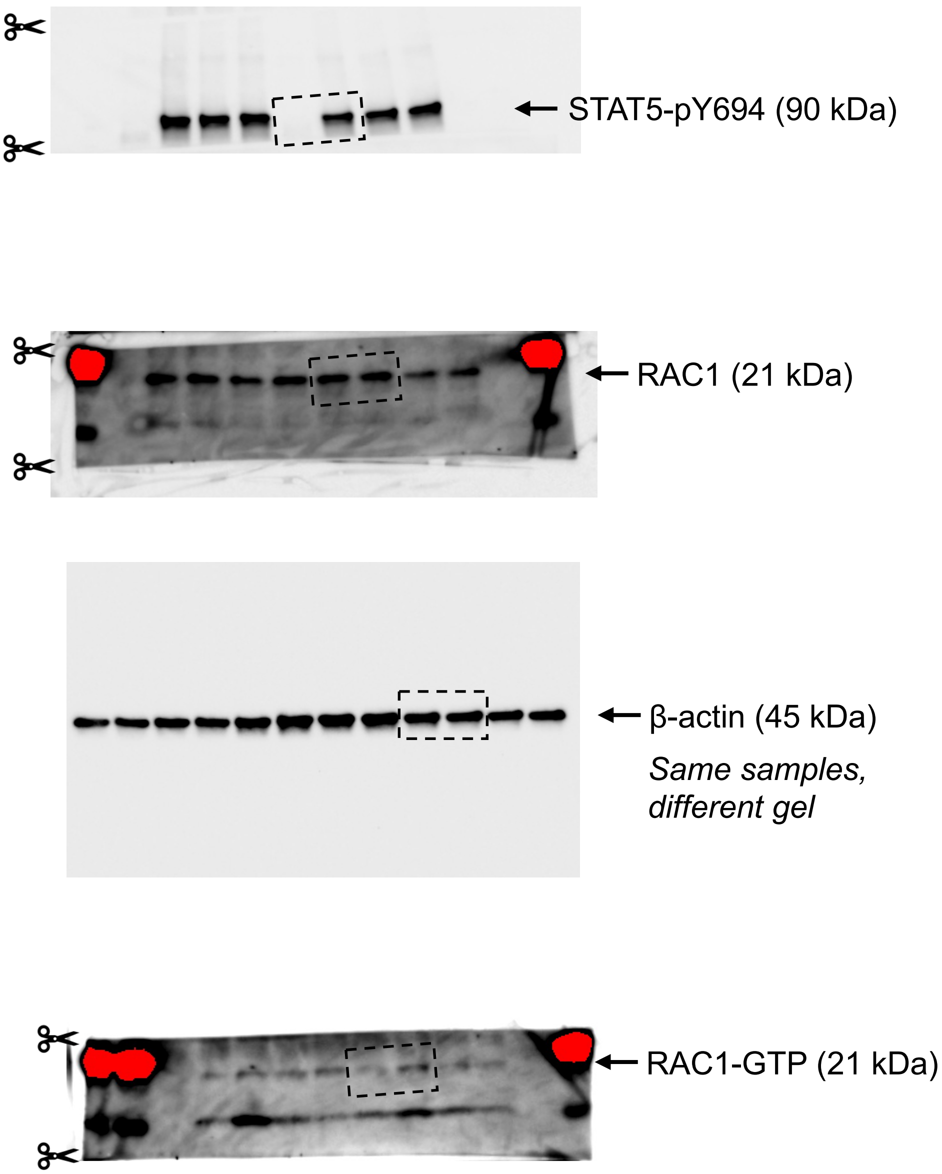

Full unedited gel for Figure 2B

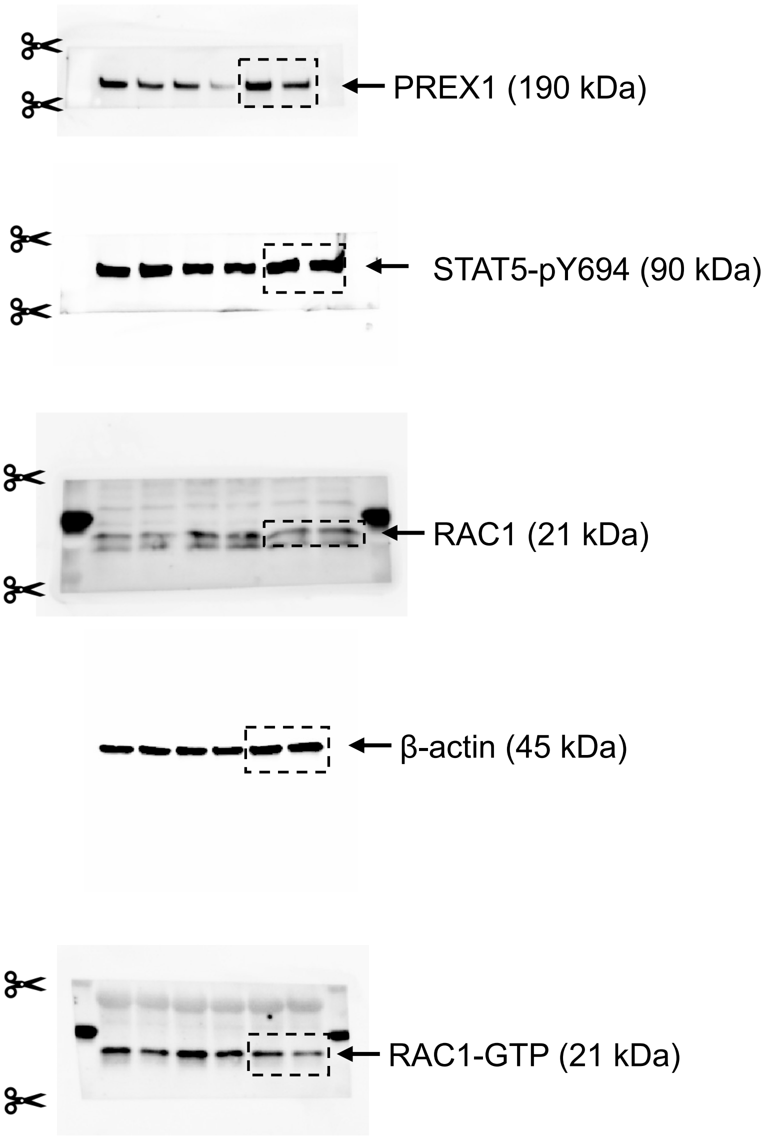

Full unedited gel for Figure 3H

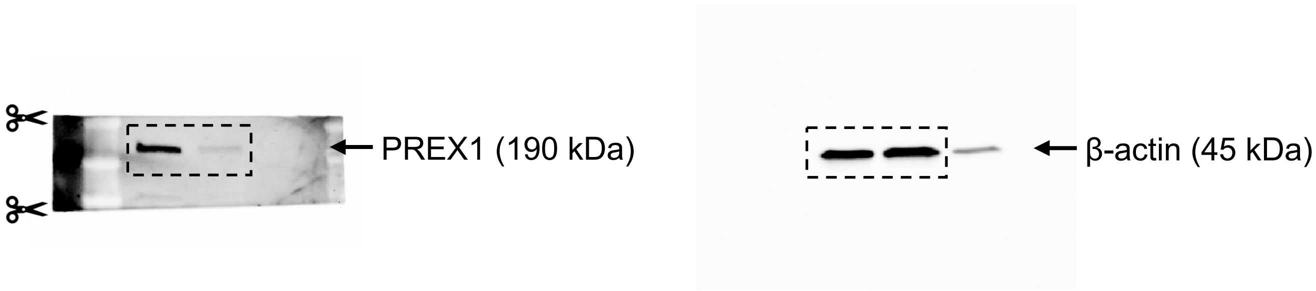

Full unedited gel for Figure 4A

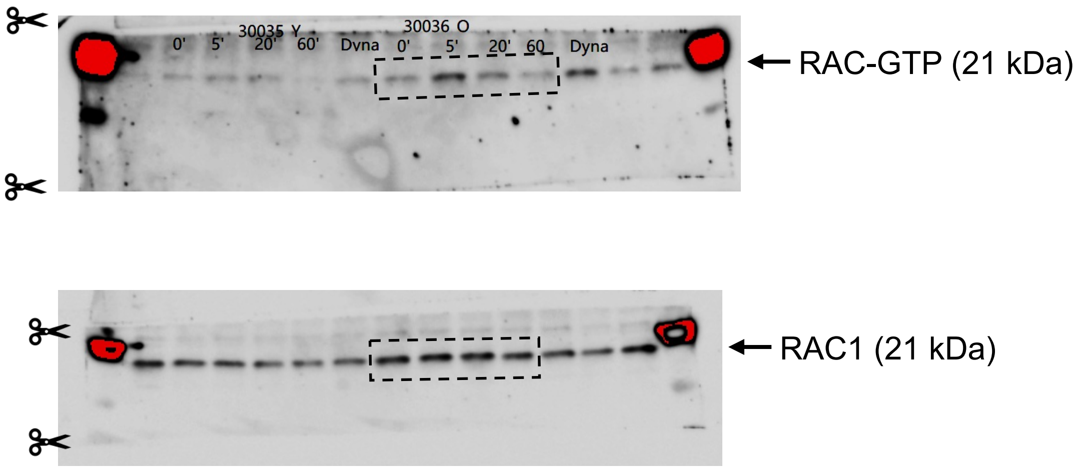

Full unedited gel for Figure 4B

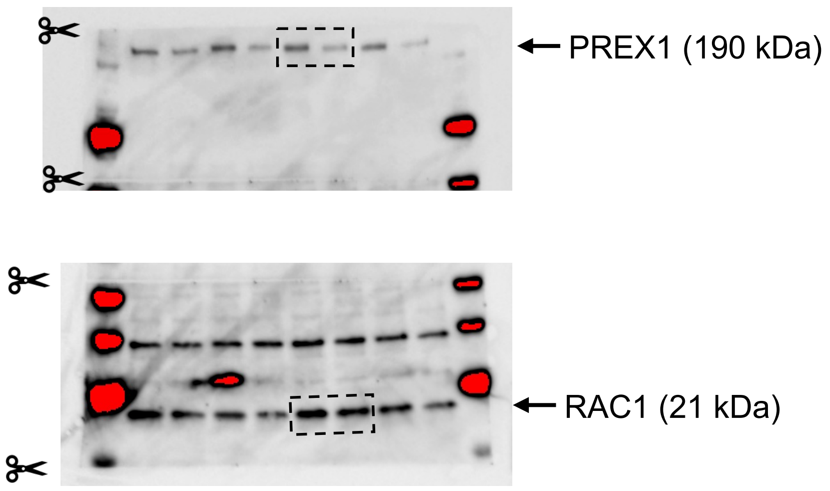

Full unedited gel for Figure S2B

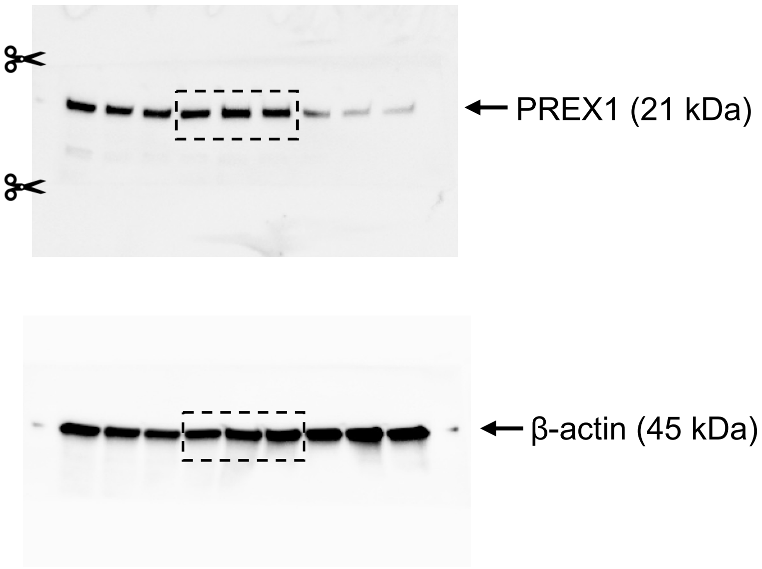

Supplement: Unedited blot and gel images [file jciinsight-9-172848-s053.pdf]
